# Axillary Reverse Mapping in Patients Undergoing Axillary Lymph Node Dissection: A Single Institution Experience From India

**DOI:** 10.7759/cureus.16462

**Published:** 2021-07-18

**Authors:** Sanghamitra Jena, Samir Bhattacharya, Arnab Gupta, Neetesh K Sinha

**Affiliations:** 1 Surgical Oncology, Tata Main Hospital, Jamshedpur, IND; 2 Surgical Oncology, Saroj Gupta Cancer Center and Research Institute, Kolkata, IND; 3 Surgical Oncology, Medica Cancer Hospital, Rangapani, IND

**Keywords:** lymphedema, breast cancer, alnd: axillary lymph node dissection, arm: axillary reverse mapping, indian population

## Abstract

Introduction

Axillary lymph node dissection (ALND) remains the gold standard for clinically node-positive and sentinel node biopsy (SLNB) positive breast cancer patients, but it is associated with the debilitating morbidity of lymphedema. Recently, a new technique of axillary reverse mapping (ARM) has been described which helps in differentiating arm lymphatics from breast lymphatics.

Aim

To evaluate the applicability of the ARM technique with blue dye and the incidence of metastases in ARM nodes in the Indian population.

Method

A total of 120 patients underwent ARM during ALND. Blue lymphatic channels and lymph nodes were noted. All axillary nodes along with ARM nodes were dissected and sent separately for pathological evaluation for metastases.

Results

ARM nodes or lymphatics were identified in 65 (54.17%) out of 120 patients. The mean ARM lymph node yield was 1.4. The patients in whom ARM lymph nodes or lymphatics were not identified had significantly higher T stage and N stage (p <0.00001) than in whom it was identified. There was no significant correlation between ARM identification with BMI, estrogen receptor (ER), progesterone receptor (PR), human epidermal growth factor receptor 2 (HER2/neu), and neoadjuvant chemotherapy (NACT) status. ARM nodes were found metastatic in three patients (7.5%). All these patients had clinically N2 disease and all had pathologically more than ten nodes involved in the axilla.

Conclusion

The identification rate of ARM nodes and lymphatics with blue dye is lower in Indian patients who present with higher clinical T and N stage disease. Other clinicopathological parameters were not associated with the identification rate. The rate of metastasis in ARM nodes is high in patients with a high axillary tumor burden. Hence, preserving ARM nodes may not be oncologically safe in higher N stage disease.

## Introduction

Breast cancer is the most common cancer in the world and, by far, the most frequent cancer among women [1]. In India, breast cancer is the most common cancer in females and the most common cause of cancer-related deaths according to Globocan 2020 [1]. 

Axillary lymph node dissection (ALND) remains the gold standard for clinically node-positive and sentinel node biopsy (SLNB) positive breast cancer patients who do not meet the criteria of the ACOSOG Z-11 trial, but it is associated with the debilitating morbidity of lymphedema [2]. A recent meta-analysis estimated that patients who underwent ALND have a lymphedema incidence four times higher than those who underwent SLNB [19.9% (95% CI: 13.5-28.2) versus 5.6% (95% CI: 6·1-7·9) respectively] [3].

Recently, a new technique has been described which helps in differentiating arm lymphatics from breast lymphatics. This technique of axillary reverse mapping (ARM) uses the injection of blue dye in the upper extremity to allow visualization and preservation of blue lymphatic channels and lymph nodes from the upper extremity during axillary lymph node dissection [4,5]. It is based on the hypothesis that the lymphatic pathway of the arm is not involved by the metastasis of the primary breast cancer and after accurately identifying and preserving the arm lymphatics, there would be no risk of lymphedema or leaving behind metastatic cells in the lymph nodes [6]. However, the identification of a group of patients in which the ARM lymph nodes and lymphatics can be preserved safely is still evolving. 

Most of the studies have been done in the West where patients present with early-stage breast cancer and have a low likelihood of axillary lymph node metastasis. There are only a few studies on ARM in the Indian population where a large number of patients present with clinically node-positive axilla and SLNB is less commonly practiced [7-9]. This prospective study was designed to evaluate the applicability of the ARM technique with blue dye and the incidence of metastases in ARM nodes in the Indian population.

## Materials and methods

This was a prospective interventional study carried out in the Department of Surgical Oncology in a comprehensive cancer hospital in Eastern India. The study was approved by our research ethics board and informed consent was taken from all patients participating in the study.

Female patients ≥18 years with pathologically proven breast cancer undergoing axillary lymph node dissection as part of their treatment were included in the study. Patients who had previous surgery of the upper limb or axilla, past history of axillary radiation, allergic to the blue dye, and pregnant or lactating women were excluded. A total of 120 patients were enrolled in the study.

**Figure 1:**
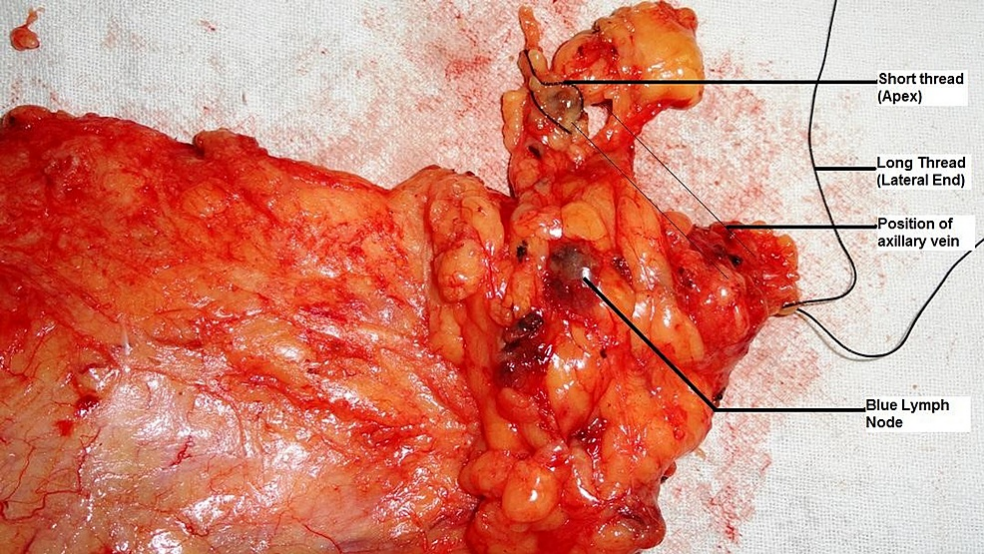
An ex-vivo picture showing Blue ARM node in relation to the axillary vein

For the axillary reverse mapping procedure, 2-4 ml of blue dye (methylene blue) was injected subcutaneously in the upper inner arm of the ipsilateral side along the medial intermuscular groove about 5cm distal to the fold of the axilla. The injection was given approximately 30 minutes before ALND, followed by massage at the injection site and elevation of the arm for 5 minutes. During axillary lymph node dissection (ALND), the blue nodes and lymphatics were searched for after the dissection through the axillary fascia (Figure 1). The patients in whom the blue nodes were identified had the blue nodes dissected out and sent separately for histopathology evaluation. These lymph nodes were evaluated with serial sectioning and hematoxylin and eosin staining to detect any metastases.

Data management and statistical analysis

Descriptive statistics were used for the calculation of mean, standard deviation, and range in normally distributed data. For skewed data, median and interquartile range was calculated. Kolmogorov-Smirnov test (K-S test) was employed for assessment of the normality of the data. Student's t-test was used in the comparison of normally distributed data, whereas for skewed data, the Mann Whitney U test was used. The chi‐square test was used to estimate intergroup differences. Two‐tailed p values <0.05 were considered to be statistically significant.

## Results

One hundred twenty patients were included in this study. The mean age of patients was 49.4 years (range 22-73 years) and the mean body mass index (BMI) was 27.53 (range 17.8-40.8). The clinical characteristics of the patients are included in Table 1.

**Table 1:** Clinical Characteristics of Patients Included in the Study

Variable	No. of patients (n=120)	%
Tumor Site		
Upper outer quadrant	46	38.33
Central	30	25
Lower outer quadrant	19	15.83
Upper inner quadrant	10	8.33
Diffuse	9	7.5
Lower inner quadrant	6	5
Received neoadjuvant chemotherapy	30	25
Clinical T stage		
T1	17	14.17
T2	63	52.5
T3	19	15.83
T4	21	17.5
Clinical N Stage		
N0	25	20.83
N1	67	55.83
N2	16	13.33
N3	12	10

Operative parameters

A total of 57 ARM nodes were identified in 40 out of 120 patients (33%). Five ALND fields were described as follows- Field A: the area between the axillary vein and the second intercostobrachial nerve, and close to the anterior edge of the latissimus dorsi muscle; Field B: the area medially adjacent to field A and close to the anterior serratus muscle; Field C: the area below the second intercostobrachial nerve and close to the anterior serratus muscle; Field D: the area below the second intercostobrachial nerve and close to the anterior edge of the latissimus dorsi muscle; Field E: the area above the axillary vein [10]. In most patients, the ARM nodes were located in field A (Table 2).

**Table 2:** Operative and histopathological parameters of patients in whom ARM node (s) / lymphatics were found

Variable	No. of patients	%
ARM Nodes (n=120)		
Identified	40	33
Not identified	80	77
ARM Lymphatics (n=120)		
Identified	58	48.33
Not identified	62	51.67
ARM Nodes / Lymphatics		
Both	33	27.50
Either of two	65	54.17
Number of ARM nodes found (n=40)		
1	24	60
2	15	37.5
3	1	2.5
Position of the ARM nodes (n=40)		
Field A	27	68.4
Field B	5	12.28
Field C	3	7
Field D	3	7
Field E	2	5.26
Position of lymphatics (n=58)		
<2 cm of axillary vein, lateral to thoracodorsal bundle	43	74.13
>2 cm of axillary vein, lateral to thoracodorsal bundle	7	12.06
<2 cm of axillary vein, medial to thoracodorsal bundle	8	13.79
Histopathology of the ARM nodes (n=40)		
Metastatic	3	7.5
Non-metastatic	37	92.5

Correlation of variables with identification of ARM nodes and lymphatics 

In the present study, the following variables were evaluated for the association with detection of ARM and lymphatic: BMI, location of the tumor, number of histologically positive axillary node, pathological T and N stage of the tumor, Estrogen Receptor (ER), Progesterone Receptor (PR), HER2/neu and neoadjuvant chemotherapy (NACT) use (Table 3, 4). We classified the patients into six groups according to the identification of ARM nodes and lymphatics for comparison with different variables. The association of BMI and location of tumor were not statistically significant with ARM nodes or lymphatics identification (p >0.05). The ARM nodes or lymphatics detected were significantly associated with the number of histologically positive axillary nodes (p <0.05). The more the positive axillary nodes, the lesser the identification of ARM nodes or lymphatics. The patients in whom ARM lymph nodes or lymphatics were not identified had significantly higher T stage than in patients where they were identified (p <0.05). A similar observation was seen when the N stage was considered. There was no significant (p >0.05) correlation of ER, PR, and HER2/neu status with ARM nodes or lymphatics identification. All patients who received chemotherapy (29) had Stage III disease except 1 patient of stage IIA. The NACT status was not associated (p = 0.48) with ARM identification among stage III patients.

**Table 3:** Variation No. of positive axillary lymph nodes in the groups

Variable	LN Not Identified (Group1)			LN Identified (Group2)			
	Median	IQR**	Range	Median	IQR**	Range	p Value
No. of Positive Nodes	8	16.25	0-38	1	5.25	0-20	<0.05
	Lymphatics not Identified (Group 3)			Lymphatics identified (Group 4)			
No. of Positive Nodes	13.5	16	0-38	0	3	0-12	<0.05
	Neither LN nor Lymphatics identified (Group 5)			Either LN or lymphatics identified (Group 6)			
No. of Positive Nodes	14	16	0-38	1	4	0-20	<0.05

**Table 4:** Correlation of ARM Nodes or Lymphatics Identification with Clinicopathological parameters

	ARM Nodes or Lymphatics identified, n(%)	ARM Nodes or Lymphatics not identified, n(%)	p Value
T Stage (n=120)			
T1	15 (88.24)	2 (11.76)	<0.00001
T2	41 (65.08)	22 (34.92)	
T3	6 (31.58)	13 (68.42)	
T4	3 (14.29)	18 (85.71)	
N Stage (n=120)			
N0	22 (88)	3 (12)	<0.00001
N1	40 (59.7)	27 (40.3)	
N2	3 (18.75)	13 (81.25)	
N3	0 (0)	12 (100)	
ER (n=120)			
Positive	43 (55.84)	34 (44.14)	0.62
Negative	22 (51.16)	21 (48.84)	
PR (n=120)			
Positive	48 (60)	32 (40)	0.07
Negative	17 (42.5)	23 (57.5)	
HER2/neu (n=120)			
Positive	9 (52.94)	8 (47.06)	0.91
Negative	56 (54.37)	47 (45.63)	
NACT in Stage III disease (n=47)			
Yes	3 (10.34)	26 (89.66)	0.48
No	3 (17.65)	14 (82.35)	

## Discussion

Lymphedema has been a dreaded complication of breast cancer surgery. SLNB, ARM, and lymphatic microsurgical preventive healing approach (LYMPHA) are surgical procedures described to prevent it. ARM is a relatively new concept under evolution since its inception by Thompson et al. over a decade ago [4]. There are only a few studies done till date and the data on the Indian population is even more sparse [11]. The preservation of ARM nodes and lymphatics has been implicated in the prevention of lymphedema. However, there are multiple queries yet to be answered for the identification of patients in which ARM can be used without compromising oncological safety. The factors for successful identification of ARM nodes and lymphatics can be tumor-related like tumor site & size, the number of metastatic axillary nodes, receptor status, etc; patient-related like BMI, etc; treatment-related like NACT, etc; and procedure-related factors like the type of dye, time interval between dye injection and surgery, experience of the operating surgeon, frequent location of ARM nodes, etc. Factors influencing metastasis in ARM nodes and identification of cross-over nodes, also need to be addressed before establishing it as a standard procedure for lymphedema prevention. 

In our study, we were able to identify the blue ARM nodes or lymphatics in 65 (54.17%) out of 120 patients. The reported identification rate of ARM node/lymphatics is in the range of 47-89% in patients where the blue dye was used and ALND was done [11]. In their prospective study, Ponzone et al. could identify ARM in 55% of patients, where 46 (94%) out of 49 had axillary nodal metastasis [12]. Bedrosian et al. had a 50% ARM identification rate in their study involving 30 patients with 3 (10%) N0 and 27 (90%) N-positive disease [13]. Forty-seven percent ARM nodes were identified by Kuusk et al. (2014) in 15 ALND cases, all had N+ disease [14]. In a study done by Ngui et al. involving 87 breast cancer patients with 38 (44%) N0 disease and 29 (33%) N+ disease, the ARM identification rate was 77% [15]. More recently, Beek et al. (2020) could identify 78% ARM nodes or lymphatics in 94 patients with half of the patients without nodal involvement [16]. The large variation of ARM identification in the reported identification can be attributed to the small sample size or heterogeneous group of the population studied. Tumor burden, NACT, and surgeon's experience are the main factors that have been implicated for the ARM identification rate using blue dye [4,12,17,18]. In our study 79% of the patients had N+ disease. This can possibly explain the lower ARM identification rate.

We identified one node in 24 (60%), two nodes in 15 (37.5%), and three nodes in one (2.5%) patient. The mean lymph node yield was 1.4 compared to the reported mean ARM nodal yield ranging from 1-2.5 in studies using dye injection [13,19-21].

The location of ARM nodes is not well studied and their classification is not uniform in the publications. We adopted the classification defined by Ikeda et al. which is most comprehensive and based on axillary vein & second intercostobrachial nerve [10]. In our study, in 68.4% of cases ARM nodes were identified in the area between the axillary vein and the second intercostobrachial nerve, and close to the anterior edge of the latissimus dorsi muscle (Field A). Likewise, Field A was the most common location (63%) in the Ikeda study [10]. Han et al. reported the most common ARM location between the inferolateral side of axillary and thoracodorsal vessels in 58.76% of individuals similar to our study [20]. Location of the ARM lymphatics and ARM lymph nodes in the majority of cases (92%) was clustered either at the level of the axillary vein or within 2 cm inferior to the vein [13]. We found the most common blue lymphatics (74.13%) within 2 cm of the axillary vein near its lateral end. Similar results were shown by Bedrosian et al. and Ikeda et al. [10,13]. The knowledge of the most common location can be vital in cases where ARM is difficult to locate.

The T stage of the disease probably affects the identification of ARM nodes or lymphatics. The patients in whom ARM lymph nodes or lymphatics were not identified had significantly higher T stage (p <0.00001) than in whom it was identified. The correlation between tumor size and ARM identification rate was reported by only a few authors. Ponzone et al. (2009) and Tausch et al. (2013) have found no correlation between ARM nodes detection rate and tumor size [12,17]. However, they have used tumor size and not T stage per se for analysis.

A similar observation was seen when metastatic nodal status was considered. The ARM identification rate inversely correlated with the number of metastatic nodes (p <0.05) and N stage (p <0.00001). In previous reports, this parameter was also not studied well. Nos et al. found two cases of negative lymphoscintigraphy where they also detected massive metastatic involvement of axillary nodes [22]. In the study of Sakurai et al., four patients in whom the lymphatics could not be detected had cancers involving the axillary lymph node and severe lymph stasis on the affected side before surgery [18]. The low ARM identification rate (47%) in a Canadian study was attributed to the extensive tumor burden of his ALND group of all 15 N+ patients [14]. The statistical significance of these findings was not tested in the above studies. However, Ponzone et al. did not find any correlation between the number of metastatic axillary nodes and the identification of ARM [12].

BMI (obesity) is an independent risk factor for lymphedema post-surgery [23]. Here we studied this factor for its association with ARM detection and found no significant correlation with it. The reported studies had similar results [12,18]. 

ER (p = 0.62), PR (p = 0.07) and HER2/neu (p = 0.91) status was not significantly associated with ARM detection. This result was in concordance with a previous study by Ngui et al. [15]. 

The indications of ALND and hence ARM preservation is overlapping with the need for NACT in breast cancer patients. In the available literature, there is no significant difference in the identification rate of ARM between the NACT group and the non-NACT group [12,17]. Hence, we included this group of patients in our study. Twenty-nine out of 30 patients who received NACT had stage III disease. As T and N stage are independent factors associated with ARM identification, we compared only stage III patients to eliminate confounding bias. In these stage patients, there was no significant difference (p = 0.48) in ARM identification.

Metastasis to ARM nodes is the most important issue from the oncological safety point of view. In our study, ARM nodes were found to be metastatic in three patients (7.5%). All these patients had a high tumor burden and a similar location of the node - 2cm inferior to the axillary vein, lateral to the thoracodorsal pedicle. All these patients had clinically N2 disease and all had pathologically more than ten nodes involved in the axilla. The ARM metastasis rate was ranging from 0-31 % in previous studies [11]. Researchers have found a significant positive correlation between metastatic involvement of ARM node with axillary tumor burden in terms of the number of positive axillary nodes [12,22,24,25]. The probable explanation of involvement of ARM node in patients with high tumor burden is the alteration of lymph flow and opening up of new channels in case of clogging of lymphatics with the tumor cells.

In our study, we tried to address the feasibility of axillary reverse mapping with blue dye, in patients in the Indian scenario where a large number of patients present with locally advanced disease. In the present prospective study, we could infer that ARM nodes were difficult to identify as the tumor and node stage of the disease progressed. The metastatic ARM nodes may be associated with a heavy axillary tumor burden. While we tried to correlate the ARM identification rate with BMI, T and N stage of disease, location of tumor, ER/PR, HER2/neu status, and NACT status systematically, which were not well evaluated in previous studies, a relatively small sample size and heterogeneous population were limitations of our study. This study will enrich the database of ARM, especially in the Indian scenario. 

As breast cancer treatment is evolving, the indications of complete axillary surgery are decreasing. However, conventional ALND remains a standard treatment for the majority of node-positive cases, which increases the importance of ARM identification and preservation to prevent lymphedema. Larger randomized controlled trials (RCTs) involving sentinel lymph node biopsy, tumor size, positive axillary nodes, crossover nodes, histopathological parameters are needed to identify which set of patients will be benefited most without compromising oncological safety.

## Conclusions

The ARM is an innovative technique to identify and preserve the lymphatics and nodes draining arm. This will prevent morbid lymphedema post-ALND. We found a low identification rate of ARM nodes in the advanced T and N stage using blue dye. Other clinicopathological parameters were not associated with the identification rate. The rate of metastasis in ARM nodes is high in patients with a high axillary tumor burden. Hence, preserving ARM nodes may not be oncologically safe in higher N stage disease. However, larger studies are needed to investigate whether combining other factors will help identify the patients who will benefit from this novel technique.
